# Extensive Identification of Bacterial Riboflavin Transporters and Their Distribution across Bacterial Species

**DOI:** 10.1371/journal.pone.0126124

**Published:** 2015-05-04

**Authors:** Ana Gutiérrez-Preciado, Alfredo Gabriel Torres, Enrique Merino, Hernán Ruy Bonomi, Fernando Alberto Goldbaum, Víctor Antonio García-Angulo

**Affiliations:** 1 Departamento de Microbiología Molecular, Instituto de Biotecnología, Universidad Nacional Autónoma de México, Cuernavaca, Morelos, México; 2 Department of Microbiology and Immunology, Sealy Center for Vaccine Development, University of Texas Medical Branch, Galveston, Texas, United States of America; 3 Department of Pathology, Sealy Center for Vaccine Development, University of Texas Medical Branch, Galveston, Texas, United States of America; 4 Fundación Instituto Leloir, IIBBA-CONICET, Buenos Aires, Argentina; 5 Centro de Genómica y Bioinformática, Universidad Mayor, Campus Huechuraba, Santiago, Chile; Wilfrid Laurier University, CANADA

## Abstract

Riboflavin, the precursor for the cofactors flavin mononucleotide (FMN) and flavin adenine dinucleotide, is an essential metabolite in all organisms. While the functions for *de novo* riboflavin biosynthesis and riboflavin import may coexist in bacteria, the extent of this co-occurrence is undetermined. The RibM, RibN, RfuABCD and the energy-coupling factor-RibU bacterial riboflavin transporters have been experimentally characterized. In addition, ImpX, RfnT and RibXY are proposed as riboflavin transporters based on positional clustering with riboflavin biosynthetic pathway (RBP) genes or conservation of the FMN riboswitch regulatory element. Here, we searched for the FMN riboswitch in bacterial genomes to identify genes encoding riboflavin transporters and assessed their distribution among bacteria. Two new putative riboflavin transporters were identified: RibZ in *Clostridium* and RibV in *Mesoplasma florum*. Trans-complementation of an *Escherichia coli* riboflavin auxotroph strain confirmed the riboflavin transport activity of RibZ from *Clostridium difficile*, RibXY from *Chloroflexus aurantiacus*, ImpX from *Fusobacterium nucleatum* and RfnT from *Ochrobactrum anthropi*. The analysis of the genomic distribution of all known bacterial riboflavin transporters revealed that most occur in species possessing the RBP and that some bacteria may even encode functional riboflavin transporters from two different families. Our results indicate that some species possess ancestral riboflavin transporters, while others possess transporters that appear to have evolved recently. Moreover, our data suggest that unidentified riboflavin transporters also exist. The present study doubles the number of experimentally characterized riboflavin transporters and suggests a specific, non-accessory role for these proteins in riboflavin-prototrophic bacteria.

## Introduction

Riboflavin, or vitamin B2, is essential for all organisms. This metabolite has an important role in oxidative metabolism, as it comprises the precursor of flavin mononucleotide (FMN) and flavin adenine dinucleotide (FAD), cofactors required for several flavoprotein-mediated redox reactions [1,2]. In addition, riboflavin is also involved in a number of extracellular processes in bacteria, such as quorum sensing signaling, extracellular electron transfer and the establishment of symbiotic associations with plants [3–5]. While animals obtain riboflavin through their diets or interactions with symbiotic microorganisms, plants and most bacteria synthesize riboflavin *de novo*. The bacterial riboflavin biosynthetic pathway (RBP) produces one molecule of riboflavin from one GTP molecule and two molecules of ribulose-5-phosphate. This reaction requires the activities of five enzymes: GTP cyclohydrolase II, 3,4-dihydroxy-2-butanone 4-phosphate (3,4-DHBP) synthase, pyrimidine deaminase/reductase, 6,7-dimethyl-8-ribityllumazine synthase and riboflavin synthase [2]. In most bacteria, the RBP genes are clustered in operons, although the genetic organization and number of paralogs varies among species.

Some bacteria, such as *Listeria*, *Enterococcus faecalis*, *Streptococcus pyogenes* and *Treponema pallidum*, do not possess the RBP but encode transporter proteins [6,7] that facilitate riboflavin uptake from the environment. There is experimental evidence for the activity of four types of riboflavin transport systems in bacteria. RibU, initially known as YpaA, was first characterized in *Lactococcus lactis* for its ability to introduce radiolabeled riboflavin into the cell [8]. RibU binds riboflavin and other flavins and comprises the substrate-specific (S) component of a member of the energy coupling factor (ECF) family of transporters. ECF transporters are also comprised by an energizing module which can function in combination with different S components [9–11]. Recent crystal structure and molecular dynamics studies of RibU have revealed that this protein possesses six transmembrane segments. The loop between TM5 and TM6 of RibU functions as a gate for extracellular riboflavin [11,12]. RibM, also known as PnuX, is a paralog of the PnuC nicotinamide riboside transporter that mediates the import of riboflavin and roseoflavin (a toxic riboflavin analog) in the Actinobacteria *Corynebacterium glutamicum* [13] and *Streptomyces davawensis* [14,15]. In *T*. *pallidum*, RfuA, the ligand-binding component of the RfuABCD transport system, binds riboflavin [7]. This system is found in several Spirochaetes and is related to the the ATP-binding cassette (ABC) family of transport systems. We have previously described the RibN family of transporters, which facilitate the entry of riboflavin and FMN in the Proteobacteria *Rhizobium leguminosarum*, *Ochrobactrum anthropi* and *Vibrio cholerae* [16].

The identification of most bacterial riboflavin transporters has been facilitated by the presence of an FMN riboswitch in the mRNA leader sequences. The FMN riboswitch regulates the expression of riboflavin biosynthesis or transport genes by adopting alternative secondary structures in response to FMN [17,18]. The genes *ypaA* (*ribU*) in *Bacillus subtilis*, *ribM* in Actinobacteria and *ribN* in *R*. *leguminosarum* were identified based on the presence of an FMN riboswitch prior to their experimental characterization [6,16,17]. In addition, *impX* from *Desulfitobacterium hafniense* and *Fusobacterium nucleatum*, which are regulated by an FMN riboswitch, and *rfnT*, which is encoded by the RBP operons in *Mesorhizobium loti*, *Sinorhizobium meliloti* and *Agrobacterium tumefaciens*, have been predicted to encode riboflavin transporters [6]. More recently, the RibXY system was predicted to function as riboflavin transporter based on the conservation of the FMN riboswitch in Chloroflexi [19]. As observed for other micronutrients, the riboflavin supply pathways sometimes overlap in bacteria. A single species may encode both the RBP and a riboflavin importer [6,16]. However, neither the extent of such co-occurrence in bacteria nor the role of riboflavin transport in a riboflavin-proficient organism are known.

Through an extensive search for the FMN riboswitch in bacterial genomes, we identified a series of riboflavin transporter candidates. Furthermore, the results of this study provided experimental evidence of riboflavin transport for one novel and three previously uncharacterized candidates. We analyzed the phylogenetic distribution of the riboflavin transport systems in bacteria and the co-occurrence of these genes with the RBP. Our data provide new insights into the importance of riboflavin transporters in riboflavin prototrophic bacteria.

## Materials and Methods

### Bacterial strains and growth conditions

The *Escherichia coli* ∆*ribB*::*cat* was previously constructed as described and used to characterize the activity of the RibN riboflavin transporter [16]. This strain was grown on Lysogenic Broth (LB) containing chloramphenicol (20 μg/ml) at 37°C. *E*. *coli* WT is a BW25141-derivative cured of the temperature-sensitive pKD46 plasmid through overnight growth of *E*. *coli* BW25141 [20] at 37°C without antibiotics.

### Plasmid construction

To construct pIasmids carrying the putative transporter genes and the negative control, the corresponding genes were PCR amplified and ligated into the pGEM-T Easy vector (Promega). All transporter candidate genes were cloned with their native promoters and regulatory regions. The *impX* gene was amplified from *F*. *nucleatum* ATCC 23726 DNA using the primers ImpXFw (5´- AAGGGTACCGGGCCCCTTTCTCCCCAGAGTAATCC-3’) and ImpXRv (5´- AAGGAGCTCTTGACGCAAGATTGAAGGTGC-3´). The *rfnT* gene (Oant_4451) was amplified from *O*. *anthropi* ATCC 49188 (ATCC) using the primers RfnTFw (5´- AAGGAGCTCCACTGGACGAATAATATTATGGGC-3´) and RfnTRv (5’- AAGGGTACCGCTCTGGATGAAAGAGCAGGG-3’). The *ribXY* operon (Caur_0816-Caur_0817) was amplified from *C*. *aurantiacus* strain J-10-fl DNA (ATCC) using the primers RibVFw (5´- AAGGGTACCCATTCGCTTCCTCCTTCACCG-3´) and RibWFw (5´-AAGGAGCTCCGTCGCAGGTTGTGCGACGTG-3´). The *ribZ* gene was amplified from *C*. *difficile* 630 DNA (ATCC) using the primers RibZFw (5´-AAGGGTACCAGCCTTGTGATGTATTTCCACC-3´) and RibZRv (5´-AAGGAGCTCACTTATGTAGTCAGCAATAAATAGC-3´). The negative control plasmid (pLpfA) contains the regulatory region of *lpfA* from *E*. *coli* E2348/69, which was amplified using the primers LpfAFw (5´-AAGGAATTCGAACAGAAAATGTTGCGTGAGGC-3´) and LpfARv (5´-AAGAAGCTTCAGGTCAGTGCTGGATTCACC-3´).

### Riboflavin transport assays

pImpX, and pRfnT, pRibXY and pRibZ were introduced into *E*. *coli ∆ribB*::*cat* by electroporation, and positive colonies were selected on LB agar plates containing chloramphenicol (20 μg/ml), ampicillin (100 μg/ml) and low riboflavin (2.5 μM). pImpX and pRfnT transformants were readily obtained in this low riboflavin selection. In order to obtain pRibXY, pRibZ and pLpfA transformants, high riboflavin (500 μM) plates were required. After this initial selection on high riboflavin, the *E*. *coli ∆ribB*::*cat* transformants with pRibXY and pRibZ were able to grow on low riboflavin (2.5 μM) plates. However, the plain *∆ribB* and the *∆ribB* + pLpfA always required high riboflavin to grow. For the subsequent riboflavin transport assays, the WT, *∆ribB* + pRfnT, *∆ribB* + pImpX, *∆ribB* + pRibXY and *∆ribB* + pRibZ strains were grown overnight on LB broth with the corresponding antibiotics and 2.5 μM riboflavin at 37°C with shaking. In parallel, the *∆ribB* and *∆ribB* + pLpfA strains were grown overnight on LB containing the proper antibiotics and 500 μM riboflavin. Approximately 1 ml of each culture was centrifuged at 12,000 rpm in a standard table centrifuge and the pellets were washed twice with phosphate-buffered saline (PBS). The pellets were resuspended in 1 ml of PBS, and a 100-μl aliquot was used to inoculate 5 ml of modified M9 broth [21] containing the indicated riboflavin concentrations and incubated at 37°C for 21 hours with shaking. The WT strain was grown only in M9 medium without riboflavin. The optical density of each culture was assessed at 600 nm.

### FMN riboswitch identification

A total of 1133 completely sequenced bacterial genomes were used for this analysis. These sequences were downloaded from the Kyoto Encyclopedia of Genes and Genomes. The transcriptional units (monocystronic and operons) in the genomes were predicted based on the intergenic distances and the functional relationships of the protein products of contiguous genes obtained from the STRING database as previously described [22]. Subsequently, the upstream region of each putative transcriptional unit was screened for the FMN riboswitch (Rfam: RF00050) using the CMSEARCH program from the INFERNAL package [23]. The cutoff value was 51.34 bits, as this value was the lowest observed for a riboswitch that regulates riboflavin biosynthetic operons.

### Homology search

For each pair of genomes, we performed a BLAST analysis using translated sequences to identify the Bi-Directional Best Hits (BDBHs) for further riboflavin transporters identification. One prototype for each riboflavin transporter family was selected, and the BDBHs were identified and clustered based on the e-values of their hits and using the OrthoMCL program [24] using the default parameters. The following prototypes were analyzed in the present study: ImpX from *F*. *nucleatum* ATCC 23726, RfnT from *O*. *anthropi* ATCC 49188, RibZ from *C*. *difficile* 630, RibV from *M*. *florum* L1, RibM from *S*. *davawensis* [14] and RibN from *R*. *leguminosarum* [16]. For the composite transporter systems RibXY, ECF-RibU and RfuABCD, the respective substrate binding proteins RibY from *C*. *auratiacus* J-10-fl, RibU from *B*. *subtilis* [13] and RfuA from *T*. *pallidum* [7] were used in the search. After the clustering was established for each family of transporters, we performed a BLAST against the prototype. Hits that were more than 38% identical with 80% or more total sequence coverage were initially considered as orthologs. However, for some families, this threshold was individually adjusted based on the available experimental evidence, the presence of regulatory elements, synteny and operon structure.

### Phylogenetic Reconstruction

#### Global Phylogeny

A set of relevant organisms was selected based on the phylogenetic distances evaluated from the multiple sequence alignment of a set of universally distributed proteins as previously described [25]. *Ad hoc* Perl scripts were used to remove the columns in which 75% or more of the characters were gaps. The phylogenetic distances were evaluated using the PROTDIST program of the PHYLIP package (Phylogeny Inference Package version 3.6. *Distributed by the author*. *Department of Genomic Sciences*, *University of Washington*, *Seattle*), and the phylogenetic tree of the selected set of organisms was constructed using PhyloPhlAn [26]. All programs were executed using their corresponding default parameters.

## Results

### Identification of novel putative riboflavin transporter genes via a search for the FMN riboswitch (Rfam RF00050) in bacterial genomes

Genomic analyses assessing the presence of regulatory elements, such as riboswitches, positional clustering and the co-occurrence of genes, are important tools for the inference of function of uncharacterized genes [6,27–30]. In previous studies, FMN riboswitches have been exclusively identified in genes associated with riboflavin biosynthesis or transport [6,19,31]. Hence, using the CMSEARCH program from the Infernal package [23], we searched for this regulatory element in bacterial genomes to identify riboflavin transporter candidates. This way, we identified 842 FMN riboswitches in 1133 genomes. As expected, the FMN riboswitch was primarily detected in the putative 5´ untranslated region of genes belonging to the RBP or transporter genes. Table 1 lists the names or COG numbers of the genes putatively regulated through the FMN riboswitch which do not seem directly involved in riboflavin biosynthesis. The genes encoding the riboflavin transporters RibM, RibU and RibN contain the FMN riboswitch, as previously reported [6,16,17]. Consistent with previous studies [6,19], we detected an FMN riboswitch in the genes for the putative transporter ImpX and the RibXY system. This regulatory element was also identified in a putative operon carrying the genes *Spirs_0556*, *Spirs_0555*, *Spirs_0554*, and *Spirs_0553* from *Spirochaeta smaragdinae*, encoding proteins that share sequence identity and transcriptional organization with the RfuABCD riboflavin transport system of *T*. *pallidum*.

**Table 1:** Genes that conserve the FMN riboswitch in bacteria, but do not belong to the riboflavin biosynthesis pathway.

Gen or COG	Function	Reference
***ypaA/ribU***	Substrate binding protein from modular riboflavin transport system	[8–12]
***pnuX/ribM***	Riboflavin transporter	[13–15,44]
***impX***	Putative riboflavin transporter	[6]
***ribN***	Riboflavin transporter	[16]
**Spirs_0556-Spirs_0555-Spirs_0554-Spirs_0553 operon (rfuABCD homolog)**	Riboflavin-binding ABC transporter system	[7]
***ribXY***	Putative riboflavin transport system	[19]
**COG2814**	H+ antiporter-2 family protein	Putative
***mfl576***	Permease	Putative
***yaaD***	Piridoxal phosphate synthase	Putative
**CLJU_c14910**	Carbohidrate kinase	Putative

In addition to known riboflavin transporters, we identified two predicted transcriptional units putatively regulated through the FMN riboswitch that potentially encode riboflavin transporters. First, the FMN riboswitch was identified in three *Clostridium difficile* strains upstream a putative transporter belonging to the COG2814, which contains Major Facilitator Superfamily MSF 1 PFAM domains. We named this transcriptional unit *ribZ*. Furthermore, the open reading frame Mfl576, a putative permease from *Mesoplasma florum* L1, was identified as a riboflavin transporter candidate and renamed RibV. In addition, we identified the *yaaDE* operon and *CLJU_c14910*, encoding a pyridoxal phosphate synthase involved in vitamin B6 biosynthesis in *Thermaerobacter marianensis* and a putative carbohydrate kinase in *Clostridium ljungdahlii* DSM 13528, respectively, conserving the FMN riboswitch. These proteins have no obvious role in riboflavin metabolism or transport.

### Characterization of putative riboflavin transporters

**Fig 1:**
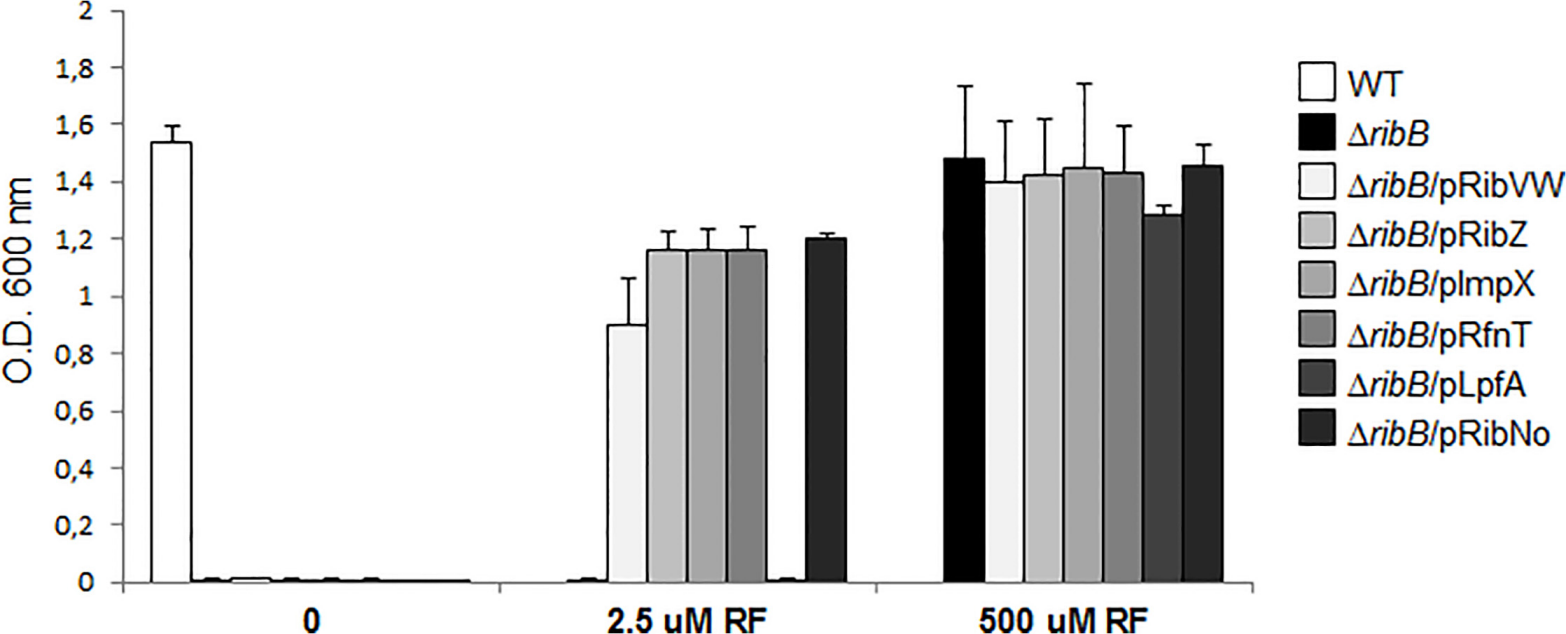
Complementation of a riboflavin *E*. *coli* auxotroph with the candidate riboflavin transporter proteins. *E*. *coli* ∆*ribB*::*cat* and derivatives bearing plasmids encoding the indicated riboflavin transporter genes and negative control were grown at 37°C on modified M9 medium containing the indicated riboflavin concentrations. After 21 hours of growth, the O.D._600nm_ was assessed. For comparative purposes, the WT strain was cultivated in M9 medium without riboflavin.

To determine whether the identified candidates are riboflavin transporters, we cloned *ribZ* from *C*. *difficile*, the *ribXY* operon from *Chloroflexus aurantiacus*, *impX* from *F*. *nucleatum* and *rfnT* from *O*. *anthropi* into plasmids and evaluated their ability to complement the growth of an *E*. *coli* riboflavin auxotrophic ∆*ribB* strain. This complementation assay excluded *ribV* from *M*. *florum* because UGA, the triplet codon for the tryptophan residue in *Mycoplasma* [32], encodes a stop codon in *E*. *coli*. RibV possesses 5 tryptophan residues that would result in a truncated protein if the *M*. *florum* gene were expressed in *E*. *coli*. As previously reported [16], the *E*. *coli* ∆*ribB* is unable to grow in M9 minimal media in the absence of riboflavin because this strain bears a deletion in the gene encoding 3,4-dihydroxy-2-butanone-4-phosphate synthase, essential for riboflavin biosynthesis (Fig 1). Low riboflavin media (2.5 μM) did not support the growth of this strain, reflecting the lack of riboflavin transporters in *E*. *coli*. Hence, this strain requires large amounts of exogenous riboflavin (500 μM) to support adequate growth. The four plasmids encoding the candidate riboflavin transporters, pRibXY, pRibZ, pImpX and pRfnT, restored the growth of *E*. *coli* ∆*ribB* in low riboflavin in a similar fashion to that observed with the plasmid encoding RibN from *O*. *anthropi*, a characterized riboflavin transporter [16]. By contrast, the negative control plasmid pLpfA failed to promote the growth of the strain at low riboflavin concentration. Neither of the plasmids promoted the growth of the strain in media without riboflavin, demonstrating the riboflavin dependence of the rescue phenotype. Thus, these results indicate that RibXY, RibZ, ImpX and RfnT facilitate the uptakte of riboflavin into bacterial cells.

### Distribution of bacterial riboflavin transporter genes

**Fig 2:**
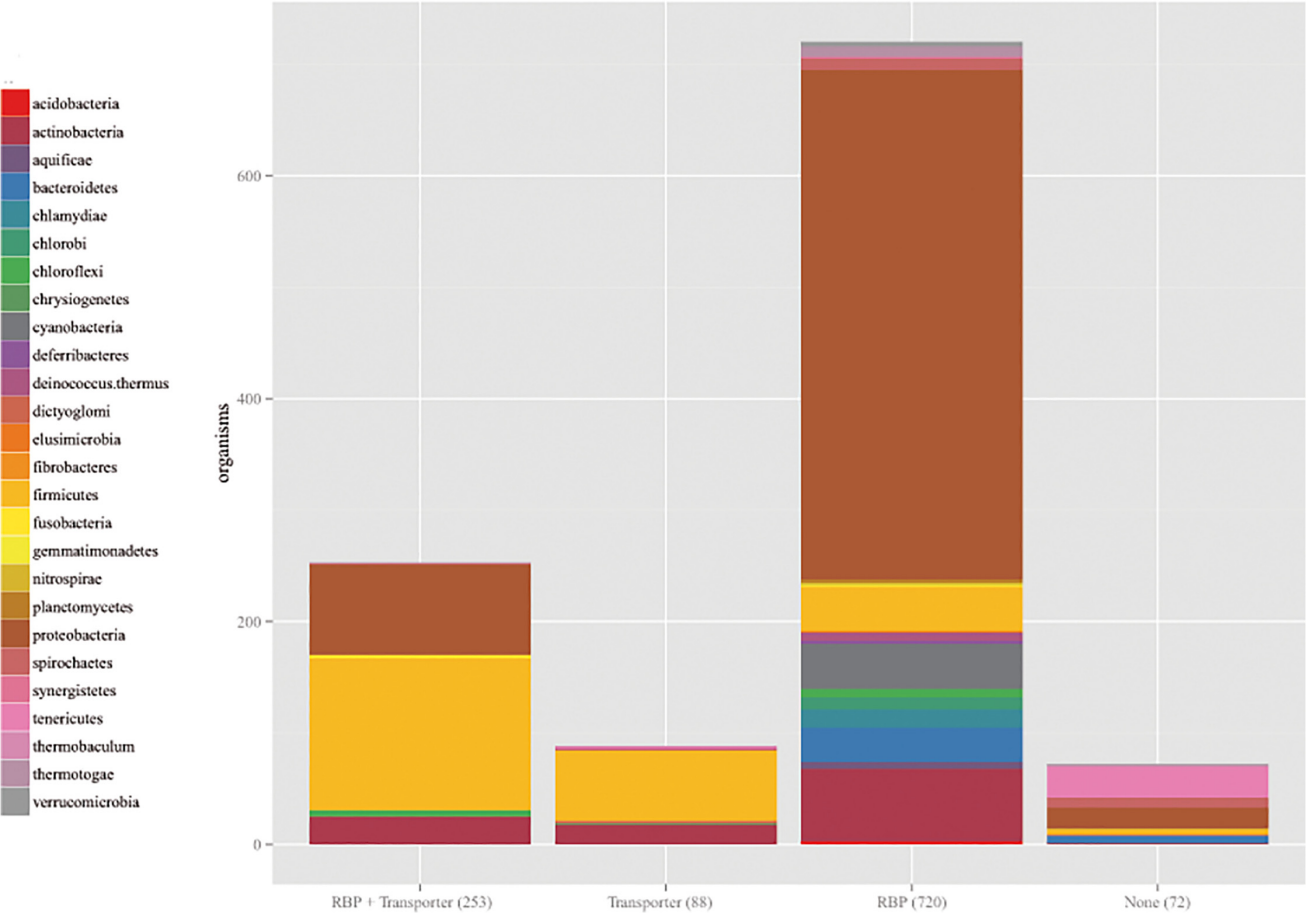
Co-occurrence of genes encoding the riboflavin biosynthetic pathway and riboflavin transporters in bacteria. Genes encoding orthologs of all known families of riboflavin transporters and representatives of the RBP were searched in bacterial genomes as described in the Experimental procedures section.

**Fig 3:**
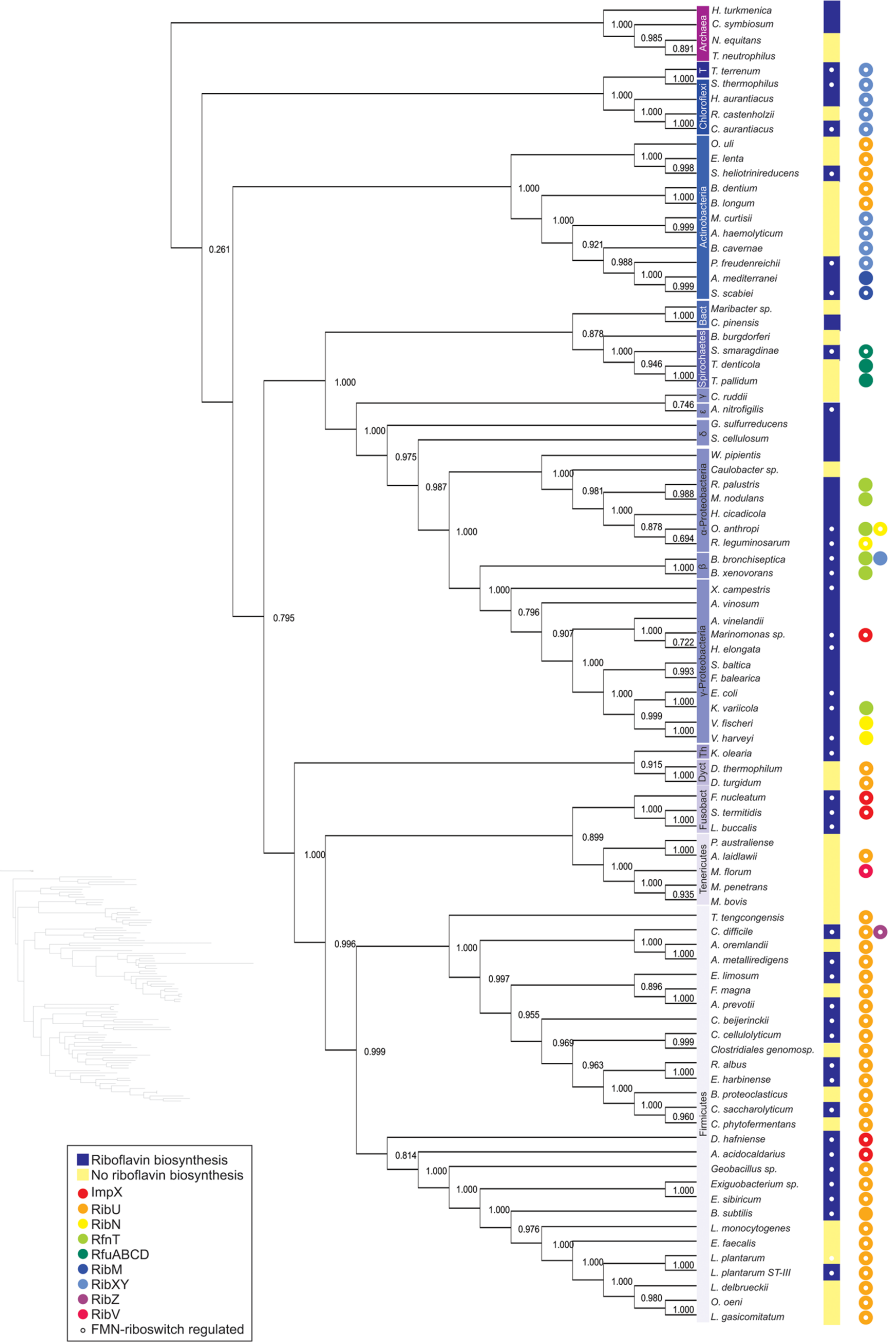
Phylogenetic tree of representative bacteria with riboflavin transporters. The organisms were randomly selected based on phylogenetic distances, and whole genomes were used as input to reconstruct the phylogeny using PhyloPhlan. Because of the length of symbiotic branches, phylogram is presented only as a preview. A yellow bar at the right of the name of the organism indicates that the bacterium does not synthesize riboflavin. A blue bar indicates species that can synthesize riboflavin. The criterion for riboflavin production was the presence of genes belonging to the COG0054 (*ribH* in *Bacillus subtilis*, *ribE* in *E*. *coli*) and COG0307 (*ribE* in *B*. *subtilis* and *ribC* in *E*. *coli*), which encode the last two steps in the riboflavin biosynthetic pathway. Phlum T = Thermobaculum. The colored circles represent orthologs of riboflavin transporters, and the white circles in biosynthesis or transport indicate the presence ot the FMN riboswitch in the putative transcriptional unit.

To gain insight into the evolution of bacterial riboflavin transporters and the association of these genes with the RBP, we determined the presence of all riboflavin transporter homologs in bacterial genomes. We selected a prototype transporter from each family and generated a list of the *bona fide* orthologs of these prototypes from 1133 bacterial genomes as described in the Materials and Methods. In addition, we also searched for the presence of RBP genes. Among the analyzed genomes, 341 (30%) contained at least one riboflavin transporter ortholog, most of which (253) also contained the RBP (Fig 2 and S1 Dataset). In 88 bacterial species, the riboflavin transporter ortholog was the sole source of riboflavin (Fig 2 and S2 Dataset). In addition, we identified 72 bacteria that did not possess either the RBP or any of the known riboflavin transporter systems (S4 Dataset) and thus likely encode unknown families of riboflavin transporters whose expression is independent of the FMN riboswitch. The results of the present study suggest that most bacteria that use a riboflavin transporter also synthesize riboflavin *de novo*. A phylogenetic analysis of representative bacteria encoding riboflavin transporters and the taxonomic distribution of each family revealed riboflavin transporters in 9 bacterial lineages (Fig 3 and Table 2). According to the distribution of the transporters, they are divided in three groups: transporters conserved across phyla, transporters restricted to one phylum and transporters restricted to a single species.

**Table 2:** Distribution of riboflavin transporters in bacterial phyla.

	*ribXY*		*ribU*		*impX*		*ribN*		*rfnT*		*ribZ*		*ribV*		*rfuABCD*		*ribM*	
RBP	+	-	+	-	+	-	+	-	+	-	+	-	+	-	+	-	+	-
Actinobacteria	o	o	o	o													o	
Chloroflexi	o	o																
Proteobacteria	o				o		o		o									
Firmicutes			o	o	o						o							
Fusobacteria					o													
Thermobaculum	o																	
Tenericutes			o	o										o				
Dictyoglomi			o	o														
Spirochaetes															o	o		

### Transporters conserved across phyla

The most widespread riboflavin transporters were RibXY, RibU and ImpX. Species from Actinobacteria, Chloroflexi and Thermobaculum possess the *ribXY* genes with the FMN riboswitch and were considered RibXY orthologs (Fig 3 and S5 Dataset). However, different regulatory elements are present in homologs of this system in other phyla. For example, the *ykkC-yxkD* element (Rfam RF00442) is present in some Cyanobacteria and β-Proteobacteria homologs. A methionine T-box (Rfam RF00230) is conserved upstream the *Lactobacillus plantarum* homolog and a thiamine riboswitch (Rfam RF00059) is present in some *rib*XY homologs in α-, δ-, and γ-Proteobacteria. Hence, this versatile transporter family could have evolved to perform riboflavin uptake in some organisms but methionine or thiamine uptake in others. In most cases, the *ribX* gene is putatively co-transcribed with the *ribY* gene, regardless of the substrate transported. RibX and RibY cluster with the COG0600 and COG0715, respectively. These genes are annotated as a permease and a substrate-binding protein from the ABC-like sulfonate/nitrate/taurine transport system family, respectively. Notably, in some Deinococci species, we found a monocystronic *ribY* gene with an FMN riboswitch. This suggests that in this lineage, the permease component is encoded in another transcriptional unit that is not regulated through the FMN riboswitch or is substituted for that of an analogous transport system. Interestingly, in addition to the RibXY system, *Bordetella bronchiseptica* also has an RfnT homolog and the RBP (Fig 3). A few other bacterial species also encode two different families of riboflavin transporters (see below).

For the assessment of the ECF-type riboflavin transport system, we used RibU as the indicator of the presence of the system. RibU comprises the substrate-binding component and thus provides the riboflavin specifity to the rest of the system. RibU was assigned to COG3601. This COG is widely distributed among bacteria and is also conserved in archaea. However, *ribU* homologs conserve the FMN riboswitch in Firmicutes, where it has been experimentally characterized [8,9,11,13] as well as in Tenericutes, Dictyoglomi and Actinobacteria (Table 2 and S5 Dataset). Among the Firmicutes, there is a second *ribU* homolog lacking a discernible riboswitch. This gene is associated with helicase and phosphatase genes, suggesting that this protein transports a different metabolite. Further experimental characterization is needed to assess whether this particular gene or other FMN-free *ribU* homologs are involved in riboflavin transport or encode divergent transporters.

The ImpX family comprises another highly distributed riboflavin transporter which was identified across the Proteobacteria, Firmicutes and Fusobacteria phyla. In these species, ImpX conserves the FMN riboswitch and is commonly encoded as a putative monocystronic unit (S5 Dataset). Some species, such as *Clostridium beijerinckii* (Fig 3), have both *impX* and *ribU*.

### Transporters restricted to one phylum

Our analyses show that RibN and RfnT are exclusively present in Proteobacteria (Fig 3 and Table 2). RibN orthologs were observed in α- and γ-Proteobacteria. In contrast to α-Proteobacteria, the RibN orthologs in γ-Proteobacteria lack the FMN riboswitch and most of these proteins are predicted to be encoded as monocystronic units, although some of these transporters are genetically associated with other membrane protein genes (S5 Dataset). Despite lacking the FMN riboswitch, these homologous proteins likely function as riboflavin transporters, such as the functionally characterized RibN protein in *V*. *cholerae* [16].

The original thresholds (described in the Materials and Methods section) used to identify RfnT orthologs through sequence comparison identified homologs that were not involved in riboflavin transport, and thus we used more stringent values (42% sequence identity with 90% coverage) for this family. The RfnT orthologs identified using these criteria are encoded by α- and β-Proteobacteria species. Although most of these genes appear to be monocystronic, some of the orthologs in the α-Proteobacteria seem to be encoded in operons with RBP genes and/or *nusB* (S5 Dataset), a transcription anti-terminator factor often genetically associated with RBP genes [16]. Other RfnT homologs putatively comprise operons containing genes whose involvement in riboflavin metabolism is not clear. For example, in *Dinoroseobacter shibae*, *rfnT* seems co-transcribed with genes involved in the biosynthesis of cobalamin and in *Paracoccus denitrificans rfnT* seems associated with a gluconate aldolase (S5 Dataset). Interestingly, some α-Proteobacteria species encode orthologs of both the RibN and RfnT families, such as *O*. *anthropi* and *R*. *leguminosarum* bv. trifolii (Fig 3 and S1 Dataset). *O*. *anthropi* RfnT was experimentally characterized in the assay shown in Fig 1 and the previously characterized RibN ortholog [16] was used as a positive control in the same assay. Thus, this experiment is the first to demonstrate the presence of two riboflavin transporters in the same bacterial species.

The gene encoding RfuA, the substrate binding subunit of the RfuABCD transporter system, clusters with COG1744 and is predicted to be co-transcribed with members of COG3845, COG4605 and COG1079 (*rfuB*, *rfuC* and *rfuD*, respectively) (S5 Dataset). The complete operon is widely distributed in bacteria. However, this system might transport riboflavin only in Spirochaetes, as the present analysis revealed RfuA orthologs that conserved the FMN element or presented high sequence similarity to the *T*. *pallidum* prototype only in *S*. *maragdinae* (45.77%) and *T*. *denticola* (58.41%); experimental evidence for riboflavin binding by RfuA has been obtained for this phylum [7]. In addition, the *rfu* operon seems regulated through different elements in other phyla. For example, in the Firmicutes *Bacillus brevis*, the *rfu* genes conserve an S box (an *S*-adenosyl methionine riboswitch), while in *Butyrivibrium proteoclasticus* and *Clostridium cellulolyticum* these genes have a purine riboswitch. Thus, genomic data suggest that this transport system may have evolved to utilize different substrates.

RibM (PnuX) was first proposed as a riboflavin transporter in part because of its homology to the nicotinamide riboside transporter PnuC [6]. In the present study, genes encoding both RibM and PnuC homologs clustered within the COG3201. These two transporters share relatively high similarity, making it difficult to predict the imported substrate through simple amino acid sequence inspection. Consistent with previous reports [6], the FMN-conserving members of the COG3201 family are only present in Actinobacteria and are predicted to be encoded monocistronically or in operon with riboflavin biosynthesis genes (S5 Dataset). Some Actinobacteria possess a second, FMN-free paralog, *pnuC*. A phylogenetic reconstruction with the members of the COG3201 (S1 Fig) suggests that PnuC, which is highly distributed in bacteria, may be an ancestral transporter that underwent duplication within Actinobacteria, followed by divergence of one of the paralogs to transport riboflavin instead of nicotinamide. This duplication might have been followed by the loss of the original gene in some Actinobacteria.

### Transporters restricted to a single species

The genes *ribZ* and *ribV*, encoding the two newly proposed riboflavin transporters, were the least distributed among the bacterial genomes analyzed. Although RibZ shares identity with another transporter widely distributed across bacteria (COG0047 and COG2814), the involvement of this transporter in riboflavin metabolism is uncertain because it does not possess the FMN element and the level of sequence identity is relatively low (from 23% to 35%). The *ribZ* gene is regulated by an FMN riboswitch only in three *C*. *difficile* strains (S5 Dataset). In addition, *Synthrophomonas wolfei*, another Clostridia, possesses a homologous gene that might also be a riboflavin transporter because this gene has relatively high sequence identity (49%) and coverage (99%). However, further experimental evidence is required to confirm this possibility because this gene lacks the FMN riboswitch and synteny with *C*. *difficile ribZ* is not conserved. On the other hand, RibV clusters with only three homologs in *Mycoplasma* and the *Mesoplasma florum* homolog is the only transporter possessing the FMN riboswitch. The other *Mycoplasma* homologs conserve neither the FMN riboswitch nor synteny with *ribV* of *M*. *florum*. Thus, the RibV family seems to be comprised by a single member in the bacteria analyzed.

## Discussion

The FMN riboswitch is one of the most widely distributed RNA regulatory motifs in bacteria [19]. Through a search for this element, we identified genes encoding two novel riboflavin transporter candidates, RibZ and RibV, as well as the previously proposed candidates ImpX and RibXY and the characterized transporters ECF-RibU, RibM, RibN and RfuABCD. Using heterologous complementation of a riboflavin auxotroph bacterial strain, an assay commonly used for the characterization of riboflavin transporter candidates [13,14,16], we confirmed the riboflavin transport activity of *C*. *difficile* RibZ. *C*. *difficile* is an intestinal toxigenic human pathogen [33]. The previously proposed candidates RibXY from *C*. *aurantiacus*, ImpX from *F*. *nucleatum* and the RfnT homolog in *O*. *anthropi* were also characterized. *C*. *aurantiacus* is an species found in hot springs and a model organism for phototrophic Chloroflexi [34,35]. *F*. *nucleatum* is a member of the subgingival plaque microbiota implicated in the development of periodontal diseases [36], while *O*. *anthropi* comprises an opportunistic pathogen of immunocompromised patients [37–39]. Interestingly, we also identified two genes that are apparently not involved in riboflavin biosynthesis or transport but do contain the FMN riboswitch. These are the *yaaDE* operon in *T*. *marianensis*, a thermophilic bacterium isolated from the Mariana Trench Challenger Deep [40] and *CLJU_c14910* in *C*. *ljungdahlii*, an anaerobic homoacetogen of importance in sustainable biotechnology industry [41]. The assessment of the actual function of these genes requires experimental evidence, as the conservation of the FMN riboswitch might indicate that they are incorrectly annotated or participate in riboflavin metabolism via a novel mechanism. Alternatively, the FMN riboswitch in these genes might suggest the existence of regulatory networks between the metabolism of riboflavin and other vitamins and carbohydrates in some bacteria.

In the present study, most of the analyzed transporters are encoded in bacterial species that synthesize riboflavin *de novo*. Importantly, the number of bacterial species encoding both functions might be further increased through the identification of new transporter families in the set of bacteria without obvious RBP or riboflavin transport systems (S4 Dataset). Some of these unknown transporter families might also be conserved among the 720 RBP-containing bacterial species that do not contain a known riboflavin transporter system (Fig 2 and S3 Dataset). Redundancy in riboflavin supply pathways has been documented previously [13,15,16]. Similarly, redundancy in the supply of other micronutrients such as vitamin B12 and amino acids is commonly found [30,42,43]. Nevertheless, in riboflavin as in other cases, the relevance of encoding both a transporter and a *de novo* biosynthesis pathway remains unclear. Moreover, in *C*. *glutamicum*, the RBP is sufficient to maintain physiological riboflavin levels independent of the RibM transporter [44]. Many species possess the FMN riboswitch upstream of the RBP and riboflavin transporter genes, while other species encode transporter genes as part of the RBP operon [6,13]. This suggests that these bacteria co-express riboflavin transporters and biosynthetic genes under low intracellular riboflavin levels. Notably, this picture resembles that of amino acid metabolism, in which bacteria tend to co-regulate both biosynthetic and transporter genes, regardless of the regulatory strategy selected [30]. An overview of this tendency suggests an accessory role for riboflavin transporters in riboflavin-proficient bacteria, in which transporters enable more rapid and energetically less costly replenishment of riboflavin pools. Certainly, it has been postulated that riboflavin-proficient bacteria conserve a riboflavin transporter because they would rather import riboflavin and shutt down biosynthesis when the vitamin is environmentaly available [15,45]. However, in some cases, riboflavin transporters are encoded independently of the FMN riboswitch and the RBP [16], suggesting that different regulatory pathways might exist, possibly rendering the expression of the transporter independent of the riboflavin requirements. Moreover, we identified bacteria with genes encoding at least two different riboflavin transporters in addition to the RBP. Among these bacterial species, *O*. *antrophi* was experimentally confirmed to possess two riboflavin transporters. In this strain, *ribN* is downstream of the FMN element, while *rfnT* is neither downstream of an FMN riboswitch nor encoded in the RBP operon, suggesting a differential expression of these transporters. Rapid riboflavin availability and metabolic energy savings through riboflavin transport instead of biosynthesis would be nearly universal evolutionary advantages among bacteria. However, the presence of riboflavin transporters is strikingly different in closely related bacteria, such as the soil Rhizobiales *O*. *anthropi* and *R*. *leguminosarum*, which encode RfnT and RibN, and *Brucella abortus*, which has been experimentally shown to lack riboflavin transporters [16,46]. Thus, we hypothesize that riboflavin transporters might have a non-redundant function in riboflavin-proficient bacteria. Although experimental evidence is still lacking, some facts support this hypothesis. A *ribN* null mutant of *R*. *leguminosarum* bv. viciae bearing an intact RBP is affected during the early stages of colonization of its host plant [16], and the overexpression of RibU helps RBP+ *Lactococcus lactis* overcome the oxidative stress induced by superoptimal growth temperatures [47]. Riboflavin availability in specific niches may be one of the determinants for the conservation of a riboflavin transporter in addition to the biosynthetic pathway. However, provided the distribution of riboflavin transporters among bacteria shown in our analysis, is difficult to associate any particular bacterial physiological tract with the presence of riboflavin transporters. Besides its role in redox metabolism, riboflavin is increasingly documented to be involved in extracellular processes. Recently, it was demonstrated that deletion of the bifunctional *ribBA* gene in the *Sinorhizobium meliloti*, the nitrogen-fixing bacterial symbiont of the *Medicago spp*. legume, does not cause riboflavin auxotrophy. However, this mutation affects the ability of the bacteria to secrete flavins involved in interspecies interaction, colonize host-plant roots and compete for nodulation. So, it is hypothesized that this bacterium has two partly interchangeable modules for biosynthesis of riboflavin, one fulfilling the internal need for flavins in bacterial metabolism and the other producing riboflavin for secretion [5]. This way, in some bacteria riboflavin provision may possess a modular arrangement with riboflavin uptake providing flavins for particular requirements. Riboflavin transporters could also be part of the bacterial machinery involved in environmental sensing. It has been reported that riboflavin and its derivative lumichrome activate the quorum sensing receptor LasR from *Pseudomonas aeruginosa* [3], thus, riboflavin potentially represents a signaling molecule.

The distribution of genes for riboflavin transporters such as ECF-RibU, RibXY and ImpX suggests the existence of ancestral transport systems. These transporters are conserved across phyla, while other species encode phylum-restricted transporters, such as RfnT, RibN,RfuABCD and RibM. Previously, the RibXY system was only found on Chloroflexi [19]. A study published during the preparation of this report demonstrated the ability of RibY from *C*. *auratiacus* to bind flavins [48]. Interestingly, in the same work the *ribXY* genes were found associated to a gene encoding an ATP-binding component, named *ribZ* in a few Cloroflexi species. Strikingly, although *impX* is distributed among Proteobacteria, Firmicutes and Fusobacteria, it was only detected in bacteria with the RBP (Table 2). RFT2, a distant homolog of ImpX, has been characterized as a riboflavin transporter in rats and humans [49,50], further supporting the hypothesis that ImpX is an ancestral riboflavin transporter. The selective pressure to maintain this particular transporter in riboflavin-proficient bacteria but not in bacteria that use import as their sole riboflavin source is intriguing and highlights the non-redundant, specific role of riboflavin transporters. The ability of proteins distantly related to *F*. *nucleatum* ImpX, such as RFT2, to function as riboflavin transporters in a different kingdom might also suggest that the presence of ImpX orthologs in bacteria was underestimated using the parameters considered in the present study, and thus this protein could be distributed in more phyla than depicted here.

## Conclusions

Overall, the present study experimentally confirms that RibZ, RibXY, ImpX and RfnT are riboflavin transporters, increasing the number of experimentally characterized transporters in bacteria from 4 to 8. Notably, the co-occurrence of riboflavin transporters and the RBP suggests a specific role for riboflavin transporters in bacterial metabolism and highlights the existence of FMN-independent riboflavin transporter families yet to be described. The identification of new transporter families, characterization of the regulatory cues governing the expression of FMN-free riboflavin transporters and the role of these proteins in riboflavin-proficient bacteria are major aims for future studies on this subject.

## Supporting Information

S1 Dataset List of bacteria bearing riboflavin biosynthetic pathway and genes for riboflavin transporters. (XLSX)

S2 Dataset List of bacteria bearing genes encoding riboflavin transporters and lacking the riboflavin biosynthetic pathway. (XLSX)

S3 Dataset List of bacteria bearing the riboflavin biosynthetic pathway, lacking genes encoding known riboflavin transporters. (XLSX)

S4 Dataset Bacteria lacking the riboflavin biosynthetic pathway and genes encoding known riboflavin transporters. (XLSX)

S5 Dataset Predicted transcriptional organization of genes conserving the FMN riboswitch in bacteria bearing riboflavin transporters. (XLSX)

S1 Fig Phylogenetic reconstruction of PnuC and its homologs. The Bi-directional Best Hits for RibM from *S*. *davawensis* from all Actinobacteria (light blue bar); the two γ-Proteobacteria (dark blue bar) *Salmonella enterica* and *E*. *coli*; the two Firmicutes (medium dark blue bar) *Bacillus thuringiensis* and *Brevibacillus brevis* and the Spirochaete *Brachyspira pilosicoli* (medium light blue bar) were aligned with MUSCLE (Edgar, 2004). The phylogeny was reconstructed with two methodologies. First with the PHYLIP package (Phylogeny Inference Package version 3.6. *Distributed by the author*. *Department of Genomic Sciences*, *University of Washington*, *Seattle*): the alignment was bootstrapped, the distance matrix was computed (Protdist) and the consensus tree was calculated. Additionally, the same alignment was used to reconstruct a phylogeny with Mr. Bayes under a mixed amino acid model. Both topologies resulted in the same evolutionary history: a duplication of the ancestral Nicotinamide Transporter PnuC (red bar) occurred within the Actinobacteria to yield the new Riboflavin Transporter RibM (orange bar). This event probably took place in the very beginnings of the diversification of the Actinobacteridae (subclass), since the organisms with two copies (their names shown in bold and in color) belong to distinct suborders (micrococcineae, streptomycineae and corynbacterineae) while none of the members of the other subclass (Coriobacteriales) have a RibM paralog. Concerning the FMN riboswitch regulation, in some cases it appears to be gained (i.e. in monocystronic genes) whilst in other cases the RibM became associated with the riboflavin biosynthetic operon, gaining also the co-regulation by the FMN-riboswitch. This reinforces the importance of co-regulating the biosynthetic genes with their transporters for most organisms. The subsequent loss of the Nicotinamide transporter PnuC seems more frequent than the loss of the Riboflavin transporter RibM paralog. An asterisk in the orange bar means that the homolog does not meets the criteria to be considered as RibM ortholog (at least 38% identity over an 80% coverage with *S*. *davawensis* RibM; conservation of synteny and/or being FMN-regulated). However, given the two topologies mentioned above, it is reasonable to hypothesize that they are riboflavin transporters.
(EPS)
